# A new approach to grant review assessments: score, then rank

**DOI:** 10.1186/s41073-023-00131-7

**Published:** 2023-07-24

**Authors:** Stephen A. Gallo, Michael Pearce, Carole J. Lee, Elena A. Erosheva

**Affiliations:** 1 American Institute of Biological Sciences, Washington D.C., United States; 2 Department of Statistics, University of Washington, Seattle, United States; 3 Department of Philosophy, University of Washington, Seattle, United States; 4 School of Social Work, University of Washington, Seattle, United States; 5 Center for Statistics and the Social Sciences, University of Washington, Seattle, United States

**Keywords:** Peer review, Research funding, Rating and ranking

## Abstract

**Background:**

In many grant review settings, proposals are selected for funding on the basis of summary statistics of review ratings. Challenges of this approach (including the presence of ties and unclear ordering of funding preference for proposals) could be mitigated if rankings such as top-k preferences or paired comparisons, which are local evaluations that enforce ordering across proposals, were also collected and incorporated in the analysis of review ratings. However, analyzing ratings and rankings simultaneously has not been done until recently. This paper describes a practical method for integrating rankings and scores and demonstrates its usefulness for making funding decisions in real-world applications.

**Methods:**

We first present the application of our existing joint model for rankings and ratings, the Mallows-Binomial, in obtaining an integrated score for each proposal and generating the induced preference ordering. We then apply this methodology to several theoretical “toy” examples of rating and ranking data, designed to demonstrate specific properties of the model. We then describe an innovative protocol for collecting rankings of the top-six proposals as an add-on to the typical peer review scoring procedures and provide a case study using actual peer review data to exemplify the output and how the model can appropriately resolve judges’ evaluations.

**Results:**

For the theoretical examples, we show how the model can provide a preference order to equally rated proposals by incorporating rankings, to proposals using ratings and only partial rankings (and how they differ from a ratings-only approach) and to proposals where judges provide internally inconsistent ratings/rankings and outlier scoring. Finally, we discuss how, using real world panel data, this method can provide information about funding priority with a level of accuracy in a well-suited format for research funding decisions.

**Conclusions:**

A methodology is provided to collect and employ both rating and ranking data in peer review assessments of proposal submission quality, highlighting several advantages over methods relying on ratings alone. This method leverages information to most accurately distill reviewer opinion into a useful output to make an informed funding decision and is general enough to be applied to settings such as in the NIH panel review process.

## Background

### Current practice and challenges in peer review

Many advances in biomedical knowledge and development of therapeutic treatments in the last 75 years rest on the bedrock of public research funding [1]. Funding is often distributed in the form of short-term grants for which scientists compete by submitting proposals, which are in turn often reviewed by their peers and evaluated for the most meritorious and scientifically fruitful ideas [2, 3]. To do this, research funders have a variety of evaluation mechanisms. A 2013 study compiled a list of the 55 world’s largest funders [4]. Among the largest, ordered by the amount allocated, are the US National Institutes of Health (NIH) [5], the European Commission [6], the United Kingdom Medical Research Council [7], the French National Institute of Health and Medical Research (INSERM) [8], the US Department of Defense [9], the UK-based Wellcome Trust [10], the Canadian Institutes of Health Research [11], the Australian National Health and Medical Research Council (NHMRC) [12], the US-based Howard Hughes Medical Institute (HHMI) [13], the German Research Foundation (DFG) [14] and the National Natural Science Foundation of China (NSFC) [15]. Of these, 64 percent clearly used a review process in which experts discussed and scored all of the qualifying proposals at a meeting (online or face-to-face). Other mechanisms included reviews of individual proposals (where independent evaluations are generated for a specific project) as well as the utilization of research boards, where sometimes a sub-set of qualifying applicants present their ideas directly to the board.

For funders that use panel review, like the NIH and the American Heart Association (AHA), peer reviewers are often asked to rate each proposal on a numeric scale, independently scoring proposals based on specific review criteria [16, 17]. However, the resulting scores from peer review ratings can depend on epistemological differences [18], personal preference [19] and varying levels of reviewer leniency or harshness [20]. Low inter-rater reliability is a long-standing concern for interpreting peer review results ([21–24]) although a recent study suggests that this measure is “not straightforward to use in practice in a typical peer review setting, and can be misleading” in assessing the quality of peer review ([25]). Moreover, some research suggests reviewer experiences can reduce disagreement in some instances ([26, 27]). However, even if one ignores reviewer characteristics that may influence their rating, the guidance for such rating systems often includes a great deal of ambiguity, allowing participants to produce a result that may have multiple interpretations. For instance, the NIH scoring guidance for research applications indicates that for an overall impact rating of 5, this could mean either that the application, if funded, “may make a contribution of high importance to the field, but weaknesses bring down the overall impact” OR that it “may make a contribution of moderate importance to the field, with some or no weaknesses [28].” Thus, the meaning of an individual rating of a proposal relative to it’s estimated worthiness of funding is not always completely clear, which is further muddied when incorporated with other reviewers’ ratings as a summary statistic and used as a basis for funding priority.

When rating the quality of a proposal, reviewers are also generally instructed to assess the likelihood a research project will advance a scientific field in an impactful way, and to consider weaknesses that may affect this likelihood [28]. This process often forces reviewers to penalize a project based on a series of identified but minor weaknesses, despite the fact that they may find the application overall to be potentially significant. Indeed, some reports suggest listed weaknesses in the text of reviewer’s critiques are better correlated with overall scores than that of listed strengths [21]. Thus, it may be that the rating process is not capturing a complete picture of a reviewer’s evaluation of a given proposal. The difficulty in interpreting these individual ratings as well as the summary statistics of the panel ratings (often used as the priority order) and translating them into a funding priority is problematic for research funders, especially when distinguishing between similarly rated proposals, especially for projects of high quality, where only a few minor weaknesses can make the difference between a funded project and a rejection. In fact, given current budgetary limitations, agencies find themselves pressed more and more to do just this [29]. Moreover, the lack of estimates of uncertainty in the scientific priority order means funders may be tempted to re-review similarly rated proposals, introducing potentially additional sources of variability to the funding decision. An exploration of alternate approaches to this decision-making process is needed not only to provide more accuracy and consistency but also to improve the credibility of an important gatekeeping function for science, grant review, which has been under fire [30, 31].

As mentioned above, while there are a variety of peer review styles and procedures used by different funders ([32]), many peer review processes use ratings of ‘proposal quality’ by all review panelists not in conflict (NIH estimates 20-40 reviewers; [33]) to inform the final funding decisions of the next level of committee, which are based on direct comparisons of top-tier proposals (e.g. Congressionally Directed Medical Research Programs [9]). NIH’s “Advice for Reviewers” specifies that when at review panel meetings, reviewers “[d]on’t compare one application to another – they should each be evaluated independently based on the review criteria” [16]. NSF instructs reviewers “to evaluate all proposals against two criteria,” intellectual merit and broader impacts, where excellence along these criteria are not characterized as being relative to other proposals [34]. However, psychological studies across a number of areas – job performance measurement, person perception, and attitude measurement – suggest that rankings are less noisy than ratings in the sense that people are less inconsistent when they are tasked with comparing two specific objects than when they need to separately assign each object a score on a scale [35]. Some have also suggested that the use of a comparative process may improve the resolution of the peer review system [25]. Along these lines, some funders include a ranking process in assessment. For instance the Netherlands Organisation of Health Research and Development (ZonMw) instructs their commission to rate proposals based on both quality and relevance, and then rank them based on a matrix of these two scores [36]. The Canadian Institutes of Health Research (CIHR) has proposals rated by one set of reviewers, then “binned” (yes/no categories) for consideration for funding by another set of reviewers who discuss the final funding priorities at a face-to-face meeting [36]. While some limited data suggests that ranking proposals against one another in review panels may help to reduce the impact of extreme reviewers as compared to rating systems [37], none have examined combining both processes of rating and ranking proposals by the same set of reviewers to augment funding decision making.

### Ranking and Rating in Assessment

From a psychological perspective, the assessment tasks of ranking versus scoring are quite different. Research on performance and trait evaluation demonstrates that when assessing two objects along some dimension, evaluators can rank more highly the object that receives the lower score [38–41]. This happens in cases where evaluators score the objects relative to reference classes that do not explicitly include each other, and hence do not involve the direct comparisons required in ranking tasks. The same individual can arrive at what appear to be discrepant judgments about the same object: for example, a proposal may receive a good but not exceptional score and also receive a relatively high rank (top of the list), where this outcome is not psychologically inconsistent because the two evaluative tasks are different in kind.

Given the contrasting types of decision making in evaluative rating and comparative ranking, and given the discriminatory constraints of current review panels to assess proposals, it seems it would be important to capture both rating and ranking information from the same reviewer about a given proposal. Moreover, while collecting ratings provides information about the level of scientific excellence of an individual proposal relative to the goals of the funding program, and allows global comparisons of the magnitude of difference between proposals (e.g. between panels), collecting rankings allows for direct comparative statements about quality in a local sense (e.g. within a panel). Having both types of data could provide both a clearer separation between proposals in funding priority as well as an assessment on the global excellence of the science submitted for review and strives to glean as much information from a reviewer as possible to make the most informed funding decision for a given panel.

However, while the collection of rating data is already formalized at many funding agencies, the collection of ranking data is less common and thought must be given to the feasibility of the implementation strategy; for instance, some research suggests that ranking too many choices becomes difficult for assessors [42], which suggests the need for collecting partial rankings. Once collected, the data must be then aggregated and presented in an interpretable way that is useful for those making funding decisions. The statistical aggregation of ranking data is much more nuanced and involved than typical summary statistics associated with ratings, in part because there is provably no method which satisfies a collection of desirable criteria [43]. Furthermore, fitting statistical ranking models can be laborious and computationally expensive [44, 45]. Rating and ranking data must be combined to create a uniform, equitable approach across all proposals and to ensure the output is comprehensible, especially in situations where reviewers are internally inconsistent in their ratings and rankings. But this requires a sophisticated statistical method, especially in the case of incorporating partial rankings. Here we present a previously described methodology [46] for collecting both rating and ranking data from panel reviewers and for statistically modeling this information to create an ordered, funding priority list of proposals informed by both data sources. In this paper, we demonstrate how to apply this model to real data with procedures in data gathering that are relatively seamless with current review processes, and presentations of results and their interpretation that would be useful to aid funding decision makers.

## Methods

### Mallows-Binomial Model

The Mallows-Binomial model is a statistical model for identifying preferences and the level of consensus given both rankings and ratings and is described in detail in a previous publication [46]. The model was the first to jointly combine preferences from ordinal rankings and cardinal ratings into a single statistical analysis without performing data conversion. As such, the Mallows-Binomial provides a principled method for learning preferences with uncertainty from both rankings and ratings when available.

As suggested by its name, the Mallows-Binomial combines a Mallows ranking distribution with independent Binomial rating distributions. Information is shared between the two model components via shared parameters. Specifically, the model contains two parameters. The first is the vector-valued parameter $p\in [0,1]^J$, where *J* is the number of proposals. We call *p* the *integrated scores*, which we seek to estimate. Each component $p_j$, $j=1,\dots ,J$ corresponds to the perceived quality of proposal *j* on the unit interval, where values closer to 0 indicate better quality and values closer to 1 indicate lower quality. As a result, a simple ordering of the proposals via their integrated scores allows one to identify a *preference ordering* (sometimes called the *consensus ranking*). We can interpret the integrated scores as representing global comparisons among the proposals and the induced preference ordering as representing local comparisons among the proposals. As such, the model parameter *p* summarizes both global and local preferences, just as do ratings and rankings, respectively. The second parameter of interest is $\theta >0$, which we call the *consensus scale parameter*. Higher values indicate greater consensus among the rankings, while lower values indicate less consensus. The scale of this parameter may be hard to interpret and depends on both the number of proposals to be ranked and the size of the observed rankings [46]. Therefore, $\theta$ will not be directly interpreted in this work. However, consensus is still reflected in the amount of uncertainty surrounding our estimates of the integrated scores, *p*. Among panels of similar sizes, the estimated parameters may still be compared to understand relative levels of consensus. Uncertainty associated with the estimated model parameters may be estimated via the nonparametric bootstrap [47]. The assumed data-generating process and model likelihood is provided in the Appendix.

A convenient aspect of the Mallows-Binomial is its ability to handle missing data. First, missing ratings and rankings for reasons unrelated to the perception of quality of those proposals (often referred to as “missing completely at random”) may be simply ignored without biasing parameter estimation. Furthermore, the model is able to handle both complete and top-*k* rankings, where $1\le k\le J$. Furthermore, given the presence of conflicts of interest in the peer review process, the model is able to still jointly learn preferences given that different judges may be able to rank and rate slightly different sets of proposals. In such cases, the parameter $\theta$ should not be directly interpreted but the key parameter of interest, *p*, may still be used for understanding preferences.

Additionally, the model assumes that ratings and rankings are conditionally independent given the integrated scores parameter *p* and consensus scale parameter $\theta$. That is, the rankings and ratings of each judge need not be in alignment. Although this may initially seem to be an unnecessary feature of the model, internally inconsistent rankings and ratings arise frequently in practice (as seen in the case study presented in the following section). When such inconsistencies are present, other methods for jointly learning from rankings and ratings (such as the non-statistical approach presented in [48]) are not applicable. Nonetheless, this conditional independence assumption does not mean that ratings and ranking are completely independent as both of these preference measures are fully informed by the integrated scores *p* and the variability as ascribed by the Mallows-Binomial model.

The Mallows-Binomial model may be efficiently estimated using the publicly available *R* package *rankrate* [49]. For example, point estimation of model parameters took approximately 20 seconds on a standard laptop computer in the real data example presented herein, with additional uncertainty estimation requiring less than 10 minutes to complete with some parallelization. Notably, this method does not require MCMC as in some related work [50], and thus reduces the computational burden in many realistic settings. Additional technical information on the Mallows-Binomial distribution, such as the probability density function, model assumptions, goodness-of-fit tests, and estimation procedures, can be found in [46, 47]. Furthermore, we will soon implement a user-friendly interface based on the *rankrate* package to assist practitioners in using our model.

### Developing theoretical examples and modeling review data

Using the Mallows-Binomial model, we constructed several theoretical review scenarios of potential reviewer voting behavior where the approaches of (i) ratings only, (ii) rankings only and (iii) the combined model could be compared directly. In the following three toy examples, we explored the comparative usefulness of approaches (i)-(iii) in tie-breaking similarly rated proposals (example 1), dealing with partial rankings of all proposals (example 2), and robustness against reviewer inconsistencies between ratings and rankings (example 3). Finally, we carried out a case study of the panel of grant review data from the AIBS review described above, applying the Mallows-Binomial model and producing integrated scores and priority lists for the proposals to exemplify the type of output this modeling approach produces.

## Results

### Toy Examples

We provide 3 toy examples below which demonstrate the concept of the integrated score, as well as the key advantages of the Mallows-Binomial model in relation to score-only or ranking-only models.

#### Toy Example 1: Tie-Breaking Equally Rated Proposals using Rankings

The first toy example demonstrates how adding rankings may help break ties between equally or similarly-ranked proposals in a principled manner. Suppose there are 3 proposals and 16 judges, who rate each proposal using a 5-point scale (the integers between 0 and 4) and subsequently rank all proposals. Their ratings and rankings can be found in Table 1.

**Table 1:** Ratings (left) and rankings (right) from toy example 1

Proposal	1	2	3	1	2	3
Judges 1-8	0	0	3	First	Second	Third
Judges 9-16	1	1	3	First	Second	Third
Mean Rating	0.5	0.5	3			

We see that proposals 1 and 2 have the mean rating of 0.5, yet all judges prefer proposal 1 to proposal 2. Next, we display what a ratings-only model, rankings-only model, and the Mallows-Binomial model would output:

1. *Ratings-Only Model:* $\{1 = 2\}\prec 3$ on the basis of the mean ratings. There is no way of distinguishing proposals 1 and 2.
2. *Rankings-Only Model:* $1\prec 2\prec 3$ since all judges provided this same ranking. There is no method of discerning that proposals 1 and 2 are essentially tied.
3. *Mallows-Binomial Model:* Integrated scores $p=[0.125, 0.125 + 10^{-8}, 0.750]$ and induced preference ordering $1\prec 2\prec 3$.^1^ This result allows us to see both a reasonable preference order and that proposals 1 and 2 are essentially tied.

Key Takeaway: Integrated scores estimated by the Mallows-Binomial model break a tie between proposals 1 and 2 by incorporating rankings. Although the preference order provides a local comparison between objects to demarcate their quality ($1\prec 2\prec 3$), the integrated scores simultaneously suggest the global comparison that proposals 1 and 2 are essentially tied.

#### Toy Example 2: Improved Decision-Making Even with Partial Rankings

The second toy example demonstrates the practicality of the proposed method in that even partial rankings may help discern proposals accurately and reliably while minimally increasing the difficulty of assessing proposals: Given many research proposals, it can be cognitively challenging to provide a complete ranking. Furthermore, it is usually more important to make accurate distinctions between the best proposals as opposed to the worst proposals. Suppose there are 8 proposals and 16 judges, who rate each proposal using a 5-point scale (the integers between 0 and 4) and subsequently rank their top-3 proposals. Their ratings and rankings can be found in Table 2.

**Table 2:** Ratings (left) and rankings (right) from toy example 2

Proposal	1	2	3	4	5	6	7	8	1	2	3	4	5	6	7	8
Judges 1-4	0	0	1	1	2	3	3	3	First	Second	Third					
Judges 5-8	0	1	1	1	3	3	4	4	First	Second		Third				
Judges 9-12	0	1	0	0	2	2	3	4	First	Third	Second					
Judges 13-16	0	0	0	0	4	4	4	4	First	Third	Second					
Mean Rating	0	0.5	0.5	0.5	2.75	3	3.5	3.75								

We see that all judges are internally consistent and exhibit a variety of preferences. For many judges, rankings help to break ties between equally-rated proposals. On the basis of all available data, it is clear that proposal 1 is the most-preferred but the preference order of proposals 2, 3, and 4 is unclear. The remaining proposals are clearly in the bottom half and are unlikely to be funded. We now consider what a ratings-only model, rankings-only model, and Mallows-Binomial model would output:

1. *Ratings-Only Model:* $1\prec \{2=3=4\}\prec 5\prec 6\prec 7\prec 8$ on the basis of the mean ratings. There is no way of distinguishing proposals 2, 3, and 4.
2. *Rankings-Only Model:* $1\prec 2\prec 3\prec 4\prec \{5,6,7,8\}$ on the basis of the available rankings. There is no way of distinguishing proposals 5, 6, 7, and 8.
3. *Mallows-Binomial Model:* Integrated scores $p=[0.000 , 0.125, 0.125 + 10^{-8} , 0.125 + 2\times 10^{-8}, 0.438, 0.750, 0.875, 0.937]$ and induced preference ordering $1\prec 2\prec 3\prec 4\prec 5\prec 6\prec 7\prec 8$.^2^ This result allows us to distinguish proposals 2, 3, and 4 while noting that they are essentially tied.

Additionally, we display confidence-based ranking summaries for the Mallows-Binomial model and the Ratings-Only Binomial model. In the table, entries correspond to the estimated probability that each proposal is truly ranked in a given rank place. Results are calculated via the bootstrap and are limited to the first four places and first four proposals (Table 3).

**Table 3:** Probabilities of proposals in the top four rank places based on the Mallows-Binomial (left) and Ratings-Only Binomial (right) models in toy example 2

Proposal	1	2	3	4	1	2	3	4
First	1	0	0	0	1	0	0	0
Second	0	0.48	0.52	0	0	0.48	0.26	0.26
Third	0	0.52	0.47	0.01	0	0.04	0.47	0.48
Fourth	0	0	0.01	0.99	0	0.48	0.27	0.26

We draw attention to the bootstrap ranking summary for proposal 2, which seems appropriate in the joint model (approximate tie for 2nd or 3rd place) but odd in the ratings-only model (approximate tie between 2nd and 4th place, but little weight for 3rd place). This strange behavior likely stems from the ratings of judges 9-12.

Key Takeaway: Integrated scores estimated by the Mallows-Binomial and their induced preference ordering draw nuanced distinctions among proposals using both ratings and partial rankings. Specifically, the integrated scores exhibit global comparisons, such as the approximate equivalence in quality between proposals 2, 3, and 4, while the induced preference ordering clarifies the local comparison that $2\prec 3\prec 4$. Using partial rankings makes the additional ranking task cognitively easier and still allows for separation of the top proposals, which is normally the most important task for the reviewers. Furthermore, the bootstrap ranking summaries for the joint model are much more sensible since they are “anchored” by the rankings, which distinguish similarly-rated proposals.

#### Toy Example 3: Analyzing Data with Conflicting Ratings and Rankings

The third toy example demonstrates the ability of the model to appropriately capture ratings and rankings even when reviewers provide conflicting information. That is, situations in which the ranking induced by the ordering of ratings is different from the observed ranking. In real data collected by the AIBS, we frequently observe such patterns. At the same time, this example includes a small minority of judges who provide “outlier” ratings and rankings, which differ from the group and heavily influence the mean ratings.

Suppose we have 3 proposals and 16 judges, who rank all proposals and rate each using a 5-point scale (the integers between 0 and 4). Their ratings and rankings can be found in Table 4.

**Table 4:** Ratings (left) and rankings (right) from toy example 3

Proposal	1	2	3	1	2	3
Judges 1-7	0	1	3	First	Second	Third
Judges 8-14	1	0	3	First	Second	Third
Judges 15-16	3	0	3	Second	First	Third

We see that judges 1-14 (the vast majority) give essentially equal ratings to proposals 1 and 2 and rate proposal 3 far below them. However, judges 8-14 are inconsistent in that they each give proposal 1 a rating of 1 and proposal 2 a rating of 0, yet rank $1\prec 2$. Judges 15-16 think very poorly of proposal 1, however, and increase its mean rating significantly. Next, we display what a ratings-only model, rankings-only model, and Mallows-Binomial model would output:

1. *Ratings-Only Model:* $2\prec 1\prec 3$ on the basis of the mean ratings. The small minority of judges who give proposal 1 a rating of 3 heavily skew the mean ratings and thus affect the outcome.
2. *Ranking-Only Model:* $1\prec 2\prec 3$ since 14 of the 16 judges provided this same ranking.
3. *Mallows-Binomial Model:* Integrated scores $p=[0.156 , 0.156 +10^{-8} , 0.750]$ and induced preference ordering $1\prec 2\prec 3$.^3^ The integrated scores suggest that proposals 1 and 2 are essentially tied in the global sense, yet through the induced ordering appropriately suggest locally that $1\prec 2$. The outlier judges do not alter the preference ordering.

Additionally, we display confidence-based ranking summaries for the Mallows-Binomial model and the Ratings-Only Binomial model. In the table, entries correspond to the estimated probability that each proposal is truly ranked in a given rank place. Results are calculated via the bootstrap and are limited to the first three places and first three proposals (Table 5).

**Table 5:** Probabilities of proposals in the top three rank places based on the Mallows-Binomial (left) and Ratings-Only Binomial (right) models in toy example 3

Proposal	1	2	3	1	2	3
First	1	0	0	0.1	0.9	0
Second	0	1	0	0.9	0.1	0
Third	0	0	1	0	0	1

Key Takeaway: Integrated scores estimated by the Mallows-Binomial model and induced preference ordering are able to appropriately resolve judges who provide internally inconsistent ratings/rankings by recognizing that ratings of 0 and 1 for proposals 1 and 2 are essentially equal, given that 14 of the 16 judges ranked proposal 1 above proposal 2. This holds true even in the presence of two “outlier” judges who distort the mean ratings by rating proposal 1 very poorly. Additionally the joint model is more confident that $1\prec 2\prec 3$, where the ratings-only model is less confident and gives much more probability to $2\prec 1\prec 3$.

### Case Study: A panel grant review data analysis

#### AIBS Ranking Procedure

In an effort to explore the usefulness of both rating and ranking in real-world funding decisions, the American Institute of Biological Sciences (AIBS) implemented a new procedure in the review of proposals submitted to a biomedical research funding agency. In this annual competition, AIBS reviewed proposals submitted to a 2020 funding announcement describing 2 year awards that are 100-150K in budget. The historic success rates for funding hover around 10 percent. As in previous years, reviewers were recruited based on expertise levels relative to the proposals, as well as on previous review experience and diversity balance. Reviewers were given access to proposal files and evaluation forms via an online system several weeks before the panel meeting and were required to enter preliminary comments and scoring into the system in advance of a teleconference review meeting. Each application was evaluated by two reviewers in advance of the meeting, who were asked to provide a score for the overall scientific merit based on the following application criteria: Impact/Significance, Innovation, Approach, Feasibility, and Investigators/Facilities. The overall scientific merit was scored on a scale from 1 (best) to 5 (worst); one decimal place is allowed in the scores (Table 6).

**Table 6:** Scoring Definitions

Score	Adjective	Guidance
1	Excellent	Exceptionally strong with negligible weaknesses
2	Very Good	Many strengths but with some moderate weaknesses
3	Average	Some strengths but also some major weaknesses
4	Less than Average	Numerous major weaknesses and shortcomings
5	Deficient	Proposal has little or no scientific value

At the meeting, assigned reviewers presented their initial critiques to the panel, then the panel discussed (discussion is inclusive of all panelists who don’t have a conflict), and then all panelists made their final scores in the system after discussion was ended. These procedures have been the standard for the history of the program while AIBS was reviewing these proposals.

In 2020, AIBS added an additional ranking procedure to the assessment process. To collect ranking data, at the end of all proposal discussion, reviewers were provided with a link in the scoring system with a list of all the final average panel scores associated with each proposal (reviewers were blinded to any proposals where they had a conflict of interest). Thus, the list of proposals was different for each reviewer, depending on their conflicts in the review system. Reviewers were then given a link to a Google^TM^ form, allowing them to look at all the proposals on the panel and select their “top six” that they would like to see funded. The question was constrained in that only one proposal can be chosen for each ranking position (e.g. first place) and only 6 choices were allowed. It should be noted that the scoring process was not altered in any way; the ranking process occurred after all proposals were scored and access to online scoresheets were locked. Only six rankings per reviewer were collected, as the focus was on ranking projects that each reviewer deemed worthy of funding if they were allowed to choose; and it was deemed impractical to rank all of the proposals. The number of ranked proposals was determined by looking at the historical success rate for this program (3 proposals for a panel of this size) and doubling it so we could examine rankings of both proposals likely to be funded as well as those slightly farther from the funding threshold.

To create the final proposal priority list, both scores and rankings needed to be considered. As mentioned previously, while scores are important indicators of the global scientific quality relative to the goals of the funding program, the rankings are more valid for indicating local proposal quality relative to the other proposals [35]. While rankings alone can be used to determine funding priorities, as they are zero sum and allow for clear discrimination between proposals, without ratings it is not known whether any of the proposals approach the standard of excellence. If only ratings are used (as is often the case), some scores can be close or identical, making it difficult to determine priority order. In order to combine these two information sources to create a funding priority list, a statistical model was chosen to apply to the data to facilitate interpretation.

### Data Analysis

Panel 1 from the 2020 AIBS program has 12 reviewers and 28 proposals. Of the 12 reviewers, 11 were “full” reviewers and 1 was a “telecon” (TCON) reviewer, meaning he/she/they was asked to rate only 1 proposal and not rank. Ratings were provided on a 1 to 5 scale in single decimal point increments, which we have converted to the integers between 0 and 40 (a 41-point scale). Subsequently, reviewers were asked to provide a top-6 ranking.

The data have a few intricacies. First, some reviewers had conflicts of interest (COI) with one or more proposals. Specifically, one reviewer had a single COI while two reviewers each had two COI; 23 proposals had no COI while 5 each had one COI. Reviewers were not allowed to rate or rank proposals with which they had a COI. Beyond COI, some ratings and rankings were missing. There were 25 instances of missing ratings and one missing ranking among the “full” reviewers; the “TCON” reviewer provided only one rating and no ranking. In this analysis, we ensure COI missingness does not influence the likelihood of a proposal ranking and treat other missingness as missing completely at random.

**Fig. 1:**
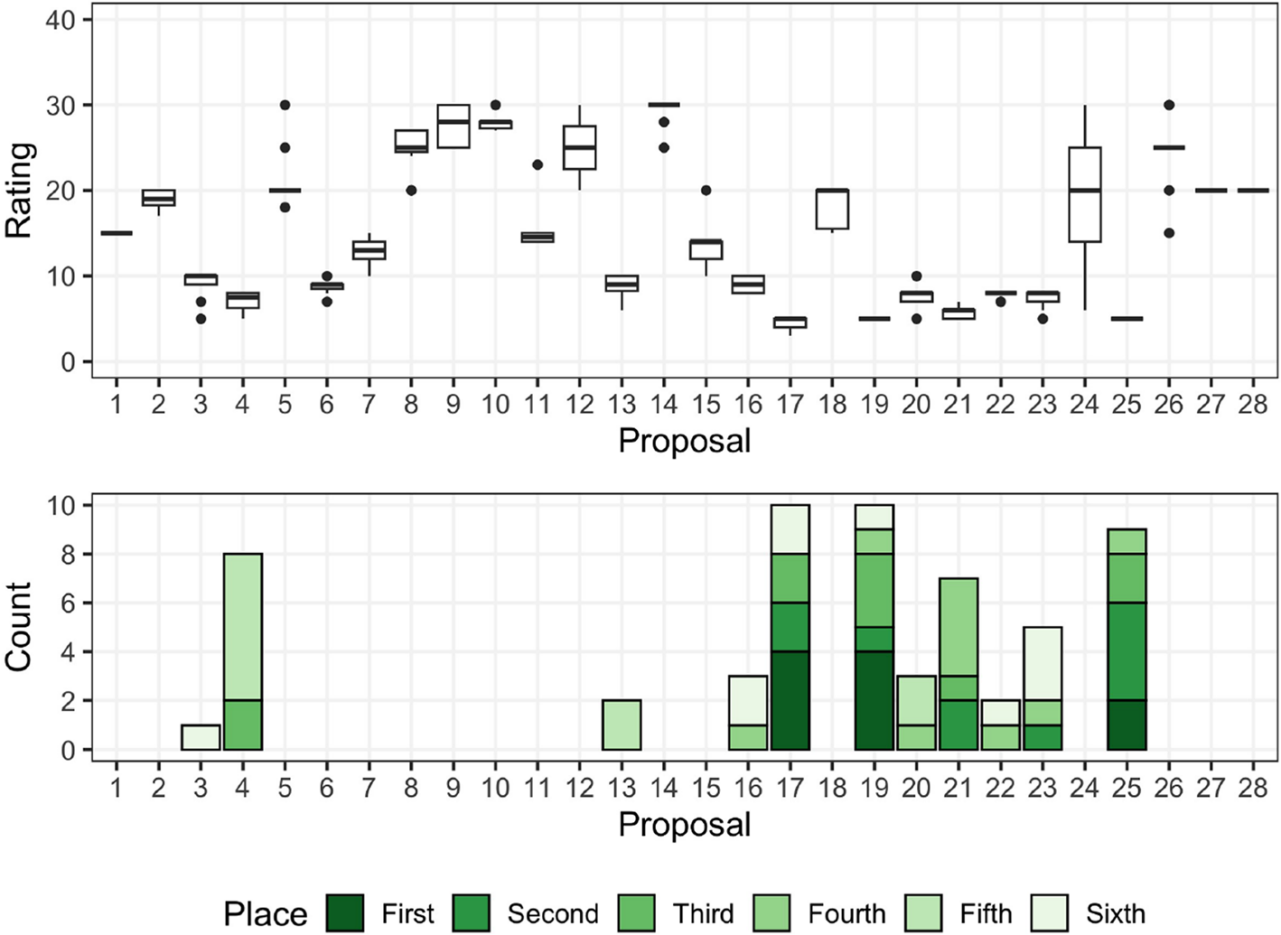
Exploratory plots for AIBS Panel 1 (2020). Top: Boxplots of scores by proposal. Bottom: Stacked barcharts of ranking places assigned across judges by proposal

Figure 1 displays exploratory plots of the ratings and rankings from this panel. We notice a variety of rating patterns among the proposals. Some proposals have consistent ratings, while others exhibit wide variance. There are a few proposals which clearly have the best ratings, while others can be immediately seen as being unlikely to receive funding. Overall, the reviewers did not use the full rating scale, instead limiting themselves to the range [3, 30], which corresponds to the range [1.3, 4] on the original scale. For rankings, we notice that only 11 of the 28 total proposals made any of the 10 provided top-6 rankings. There is no clear consensus by looking at the top few rank places. However, we see that proposal 17, 19, and 25 frequently appear in first, second, and third places; proposal 4 appears in 5th place for over half the reviewers who provided rankings.

**Fig. 2:**
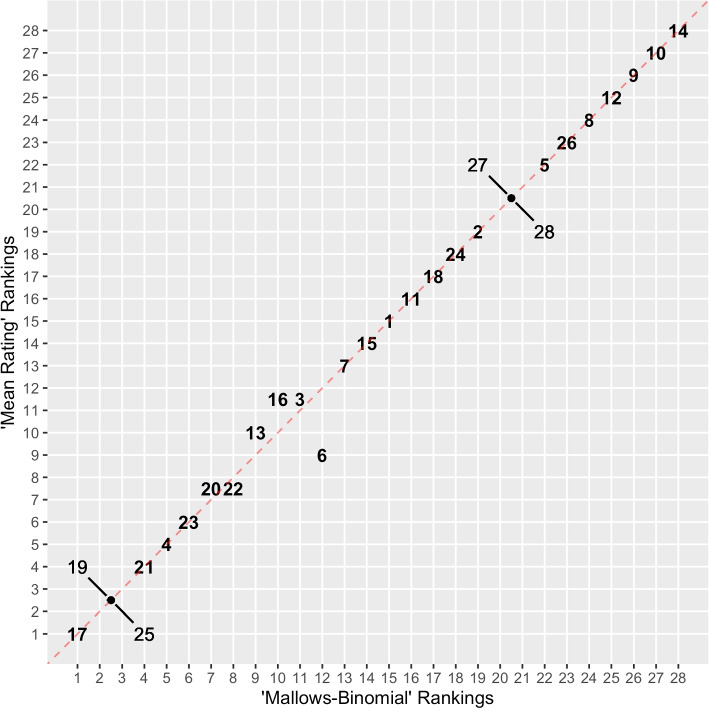
Proposals by estimated rank level in each model

We now display integrated scores estimated by applying a Mallows-Binomial (MB) model to the AIBS data. In order to draw attention to the utility of the model, we additionally provide results from a traditional method, which we call the “Mean Ratings” (MR) model. In this model, we simply take the mean ratings from each proposal and standardize them to the unit interval. The order of proposals based on their mean ratings is thus the estimated ranking of the proposals. We display results in Table 7 and Fig. 2.

**Table 7:** Estimates of integrated scores from the Mallows-Binomial (MB) and Mean Ratings (MR) models. A small positive value is added to the integrated scores of proposals 22 and 6 in order to signify that $p_{20}\prec p_{22}$ and $p_3\prec p_6$ in the MB model. The order of proposals is based on the MB model

Proposal	MB	MR	Proposal	MB	MR
17	0.114	0.114	1	0.375	0.375
19	0.125	0.125	11	0.382	0.382
25	0.125	0.125	18	0.452	0.452
21	0.143	0.143	24	0.461	0.461
4	0.175	0.175	2	0.472	0.472
23	0.182	0.182	27	0.500	0.500
20	0.195	0.195	28	0.500	0.500
22	0.195$+10^{-8}$	0.195	5	0.527	0.528
13	0.218	0.222	26	0.611	0.611
16	0.222	0.225	8	0.618	0.618
3	0.225	0.225	12	0.625	0.625
6	0.225$+10^{-8}$	0.218	9	0.690	0.690
7	0.322	0.322	10	0.703	0.703
15	0.344	0.344	14	0.731	0.731

We see in Table 7 that the estimates of integrated scores between the MB and MR models are similar. However, the preference ordering deviates in the MB model in a few cases. This distinction is made clear in Fig. 2, in which we can see directly that the MB model breaks ratings ties in 7th/8th place and 11th/12th places. Additionally, we see a reordering among proposals 3, 6, 13, and 16 between the Mean Ratings and Mallows-Binomial models: Although proposal 6 receives a slightly better mean rating than proposals 3, 13, and 16, its rankings are comparatively worse enough to make it receive a worse integrated score in the joint Mallows-Binomial model. We note that in the case of proposals 27 and 28, each received the same mean rating and neither was ranked by any judge. As such, neither model is able to break their tie. The model is also unable to break a tie in mean ratings between proposals 19 and 25. These proposals received unique rankings among the judges, yet precisely half of the reviewers preferred 19 to 25 while the other half preferred 25 to 19. As a result, the data do not allow for demarcation between these proposals on the basis of ratings or rankings. We turn to uncertainty estimation in order to make funding decisions between these two proposals.

Next, we estimate uncertainty in rank place among the proposals in Tables 8 and 9. Table 8 displays the probabilities of proposals entering first, second, third, or fourth place in the Mallows-Binomial and Mean Ratings models. In the Mallows-Binomial model, there is more certainty in proposal 19 being ranked above proposal 25, which may help us break the tie in integrated scores between the proposals. In comparison to the Mean Ratings model, the Mallows-Binomial provides more evidence that proposals 17, 19, and 25 have similarly high quality. Based on the original data, these results seem probable. For the purpose of making decisions, Table 9 displays the probabilities that proposals should receive funding conditional on the number of proposals the funding agency can support.

**Table 8:** Probabilities of proposals in each of the top-4 ranking places based on the Mallows-Binomial (left) and Ratings-Only Binomial (right) models in AIBS grant panel review data

	Mallows-Binomial					Mean Ratings			
Proposal	17	19	25	21	4	17	19	25	21
First	0.576	0.328	0.096	0	0	0.997	0.002	0.001	0
Second	0.250	0.263	0.485	0.002	0	0.003	0.499	0.498	0
Third	0.174	0.407	0.417	0.002	0	0	0.499	0.501	0
Fourth	0	0.002	0.002	0.993	0.003	0	0	0	1

**Table 9:** Probability of proposals being funded conditional on varying numbers of total proposals funded for the Mallows-Binomial model

Proposal	Fund 1 proposal	...2 proposals	...3 proposals	...4 proposals
17	0.576	0.826	1	1
19	0.328	0.591	0.998	1
25	0.096	0.581	0.998	1
21	0	0.002	0.004	0.997
4	0	0	0	0.003

## Discussion

The evaluative method described here (collecting both ratings and partial rankings) and the combination of these streams of information into one integrated score provides a coherent way to assess a group of research proposals. While funding schemas like partial lotteries embrace the seemingly stochastic nature of grant review scores and outcomes ([51]), our approach conversely works to decrease the noise in review outcomes by extracting more meaningful information from reviewers ([52]). Moreover, the output of this process is a priority list for project funding that is structured optimally for the decision type of many secondary, programmatic funding panels and offers a confidence interval or a probability surrounding a given project’s priority. This output can be catered to the expected funding levels, creating probabilities of funding preference conditional on the number of total proposals that are planned to be funded (Table 9). These features are not typically incorporated in grant review output but are crucial for making appropriate funding judgements with confidence. In addition, we show that this method allows for the usage of partial rankings of proposals by reviewers, limiting the burden on panelists, allowing for conflict of interest scenarios, and providing a practical solution for data collection by review organizers. Hesitancy at funding agencies to adopt new decision making tools may be potentially overcome by the convenience of this format, the appeal of estimates of uncertainty in the priority order as well as the availability of software to ease any burden from these computations [49], which should be further alleviated by the development of a user-friendly interface based on this software. Future work should survey funding agencies to determine their perceptions of its usefulness.

This method is likely to be especially impactful for the tie breaking of similarly rated proposals, as scores are often compressed to a limited range in peer review [53]. As in the NIH example [28], this approach may help to discriminate between projects with similar ratings but different potential importance, more so than using criteria scores as tie-breakers, as they are often correlated with the overall score [33]. As reviewer-identified weaknesses have been observed to be more closely associated with ratings of proposal quality than strengths [21], it may be that including zero sum ranking could force a more balanced decision making process by valuing these strengths; this should be a focus of future research. However, it should be mentioned that our model assumes that proposals have a true underlying level of quality, an assumption implied in peer review studies that use different proxies for proposal quality (e.g., citations, patents; [54]). Despite this common assumption, true measures of quality remain elusive.

Collecting the ranking data at the end of all proposal discussion has an added benefit, as it may help to mitigate any temporal effects, as sometimes the decision-making norms of a panel can evolve over the length of the review meeting [55, 56]. For example, the panel may reach agreement on how to interpret the review criteria only after discussion at the panel meeting [57]. The model described here could incorporate such consistent interpretation across the panel while still remaining robust to conflicts in individual reviewer ratings and rankings, as well as to outlier reviewers. Thus, this method provides for a more appropriately weighted priority list that, while often somewhat similar to lists derived from the ratings alone, is resistant to internal inconsistencies and outliers. However, given the variety of epistemological views and expertise present on most review panels ([19, 58, 59]), even when reviewers are given identical scoring instructions and allowed an opportunity to discuss these criteria during the panel meeting, there may still be subgroups of reviewers with divergent views on proposal rating and ranking. In particular, little work has explored whether reviewer preferences for sets of proposals (e.g. high risk proposals) would affect reviewer rankings; this is an area in need of further examination. While our model assumes that individual raters are using the same criteria in similar ways, this is a commonly made assumption in the inter-rater reliability literature [59]; future research should do more to explore this assumption.

## Limitations

As mentioned, research funders utilize a wide variety of peer review processes and procedures, and so the usefulness of this method will depend on this context. We have summarized the limitations and strengths of this method against common review processes in Table 10. For example, the presence of bias or strategic voting (e.g. based on connections to the applicant) impact all of these methods at some level, either directly or indirectly during the process. The addition and integration of rankings in grant review will not improve or intervene on problematic social dynamics to which scoring frameworks are already vulnerable. Like ratings, rankings are not immune from implicit bias [60, 61] or the influence of other panelists [62]. Like ratings, rankings permit reviewers to game the system by either engaging in strategically motivated voting [63] or by horse-trading favorite proposals with other panelists [19]. And, like ratings, rankings may reflect diverging interpretations of review criteria [19, 58, 59]. Rankings and our model inherit these forms of social influence, which may be inflected in the model’s results. Thus, while our integrated score provides more capability to discriminate proposals from one another, the thought process behind individual decisions is important, and still requires the use of orientations and training to counteract factors like implicit bias. It should also be noted that scores are often accompanied by written critiques, providing insight into reviewers’ motivations for their scoring decisions. More research is needed to examine whether additional guidance is needed at the ranking stage, to ensure reviewers are using similar criteria to rank proposals, or whether the allowance of different interpretations by reviewers is a feature and not a bug.

The usage of our model does have clear advantages over other methods in that it provides a tie-breaking mechanism for similarly rated proposals, of which none of the other methods are capable (although the random selection in partial lotteries avoids the issue of ties). Our model is also not easily influenced by outliers (e.g. Toy Example 3) compared to the other listed methods, especially those where only a few ratings are used to create the summary statistic that is used to determine funding. As we mention, our method is unique in utilizing more than one source of information to determine funding order and is the only method listed that directly provides a recommended funding priority list other than partial lotteries, which produce random priority lists after the initial rating phase is complete. Finally, our method provides estimates of the uncertainty in the recommended funding priority list – a feature that other review mechanisms currently lack.

It should be noted that while we list in Table 10 an example of the model being applied to a typical unblinded panel review, this type of output would even prove useful for agencies that adopt partial-lottery funding schemas, which include a proposal-rating phase ([64]). If the process contains a rating phase, the amount of extra preparation to implement our methodology would be small yet would likely provide better discrimination between proposals. Again, this method simply extracts more information from reviewers at the time of assessment and integrates it into a meaningful output, hopefully improving the interpretability of the review results, and does not abrogate the need for reviewer training, appropriate recruitment of expertise, vetting for conflicts of interest and monitoring of the process for consistency and equity. Studies have clearly shown significant strides can be made in the consistency of reviewers with even brief training programs that explain review criteria and highlight expectations from participants [65].Moreover, the generation of the proposal preference order through the use of this model does not serve as a replacement for secondary funding committees, lotteries or alternate grant allocation models that may have their own limitations ([66]). Finally, the model was applied here to review data from one panel of 28 proposals; some approximate estimation algorithms that have high accuracy can be used when there are more than 30 proposals [46].

**Table 10:** Limitations of Current Grant Review Models

Review Mechanism	Presence of Implicit Bias	Presence of Strategic Voting (based on connection to PI)	Tie Breaking Mechanism	Influence of Outlier Ratings	Sources of Reviewer Input into Recommended Funding Order	Recommended Funding Order Directly or Indirectly Determined	Level of Uncertainty in Recommended Funding Order
Unblinded Panel Review Rating of Proposal Sets	Direct	Direct	None	Medium	Rating Alone	Indirectly (Summary stats of Ratings)	None
Blinded Panel Review Rating of Proposal Sets	Indirect	Indirect	None	Medium	Rating Alone	Indirectly (Summary stats of Ratings)	None
Rating Review of Individual Projects	Direct	Direct but Only Impacting a Few Proposals	None	High	Rating Alone	Indirectly (Summary stats of Ratings)	None
Partial Lottery with Initial Rating of Proposal Sets	Initially Direct and then Random Selection	Initially Direct and then Random Selection	Initially None and then Random	High (Initial Rating with no Panel)	Initial Rating and then Random Selection	Initially Indirectly (Summary stats of Ratings) and then Randomly	Initially None and then Random
Unblinded Panel Review Rating/Ranking of Proposal Sets using Mallows-Binomial Model	Direct	Direct	Integrated Score	Low	Both Rating and Ranking	Directly	Estimated Confidence Intervals

## Conclusions

To reserach funders, the gathering of both rating and ranking data and the application of the Mallows-Binomial model to this data to generate an integrated score can have many advantages over methods relying on ratings alone. These include providing a ranked priority list with confidence metrics, a higher degree of discrimination between similarly rated proposals, and a robustness to outliers and reviewer inconsistencies. While this method may not provide a panacea for problematic forms of social influence and may need to be adapted to work well with different funding mechanisms, peer review schemas, and success rates to achieve the goals of a research funding program, it is likely that incorporating information from both local and global evaluative tasks leverages information from reviewers to most accurately distill their opinion into a useful output to make the best, most informed decision.

## Figures and Tables

**Fig. 3:**
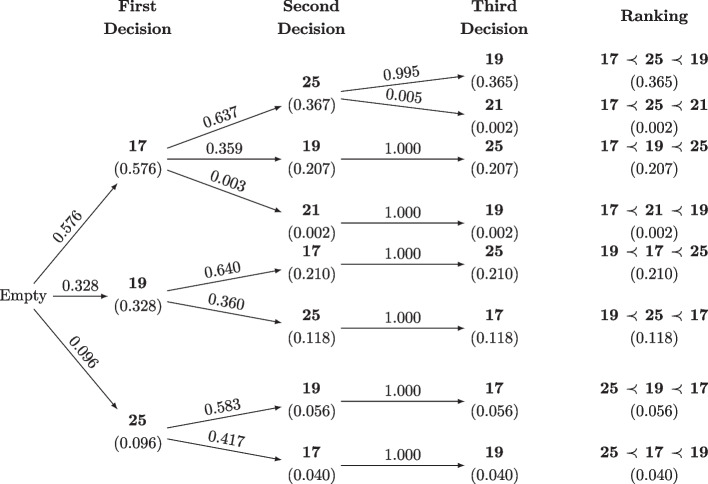
Tree diagram of proposal selection probabilities. Bolded numbers represent proposals; numbers above lines represent the conditional probability of selecting the next proposal given a current stage in the tree; numbers in circles represent joint probabilities up to that point in the tree

## Data Availability

Anonymized data sets of ranking and rating data are available at 10.6084/m9.figshare.20505747.

## Appendix

### Additional details of the Mallows-Binomial distribution

We first describe the assumed data-generating process: The Mallows-Binomial distribution assumes that each proposal, *j* has a latent quality, $p_j\in [0,1]$. By convention, values of $p_j$ close to 0 indicate better quality and values close to 1 indicate lower quality. Given $p_j$, the ratings for proposal *j* are drawn independently and identically distributed from a Binomial$(M,p_j)$ distribution, where *M* is the known maximum integer score. At the same time, a simple ordering of proposals based on their latent qualities, denoted by $\pi _0$, indicates the true order of proposals from best worst. Furthermore, the distribution assumes that the population of reviewers exhibits some level of consensus, $\theta >0$, in which low values indicate weak consensus and high values indicate strong consensus. Given $\pi _0$ and $\theta$, rankings are drawn independently and identically distribution from a Mallows$(\pi _0,\theta )$ distribution. In a Mallows distribution, $\pi _0$ represents the modal probability ranking and $\theta$ is a scale parameter that controls how likely rankings that are far away from $\pi _0$ are to be drawn. Since $p=[p_1 \ p_2 \ \dots , p_{J-1} \ p_J]$ influences both ratings and rankings, we call *p* the *integrated scores*.

We now state the model likelihood. Let $X = [X_1 \ X_2 \ \dots \ X_J]$ represent the collection of ratings assigned to proposals $j=1,\dots ,J$ by a reviewer, and $\Pi$ his/her ranking. Under a Mallows-Binomial$(p,\theta )$ distribution, their joint probability can be written:

1 $$\begin{aligned} \text {Pr}[X=x,\Pi =\pi | p,\theta ] = \prod _{j=1}^J \left( {\begin{array}{c}M\\ x_j\end{array}}\right) p_j^{x_j}(1-p_j)^{M-p_j} \times \frac{\exp ^{-\theta d_K(\pi ,\pi _0)}}{\psi (\theta )} \end{aligned}$$

In Eq. 1, $d_K(\pi ,\pi _0)$ represents the Kendall $\tau$ distance between the reviewer’s observed ranking $\pi$ and the true consensus ranking $\pi _0$. This distance may be defined as the total number of adjacent object pairs which must be swapped in the consensus ranking in order to obtain the observed ranking. $\psi (\theta )$ is a normalization constant that depends on $\theta$ and has a closed-form expression. Further details of the distribution can be found in [46].

### Additional results from case study

**Table 11:** Results from the Mallows-Binomial and Mean Ratings Models

Rank	1st	2nd	3rd	4th	5th	6th	7th	8th	9th	10th	11th	12th
MB	17	19/25	19/25	21	4	23	20	22	13	16	3	6
Ratings	17	19/25	19/25	21	4	23	20/22	20/22	6	13	3/16	3/16

## Abbreviations

AHA: American Heart Association
AIBS: American Institute of Biological Sciences
CIHR: Canadian Institutes of Health Research
CDMRP: Congressionally Directed Medical Research Programs
NIH: National Institutes of Health
NSF: National Science Foundation
ZonMw: Netherlands Organisation of Health Research and Development

## Footnotes

1 We add a small positive constant to the second value only to symbolize that $p_1<p_2$, which yields the displayed preference ordering.

2 We add a small positive constant to the third and fourth values only to symbolize that $p_2<p_3<p_4$, which yields the displayed preference ordering.

3 We add a small positive constant to the second value only to symbolize that $p_1<p_2$, which yields the displayed preference ordering.
